# Removal Characteristics of *N*-Nitrosamines and Their Precursors by Pilot-Scale Integrated Membrane Systems for Water Reuse

**DOI:** 10.3390/ijerph15091960

**Published:** 2018-09-07

**Authors:** Haruka Takeuchi, Naoyuki Yamashita, Norihide Nakada, Hiroaki Tanaka

**Affiliations:** Research Center for Environmental Quality Management, Kyoto University, Shiga 520-0811, Japan; yamashita.naoyuki.kt@ehime-u.ac.jp (N.Y.); nakada.norihide.8w@kyoto-u.ac.jp (N.N.); ick17056@nifty.com (H.T.)

**Keywords:** *N*-Nitrosamines, formation potential, membrane treatment, reverse osmosis, membrane fouling, pilot-scale

## Abstract

This study investigated the removal characteristics of *N*-Nitrosamines and their precursors at three pilot-scale water reclamation plants. These plants applies different integrated membrane systems: (1) microfiltration (MF)/nanofiltration (NF)/reverse osmosis (RO) membrane; (2) sand filtration/three-stage RO; and (3) ultrafiltration (UF)/NF and UF/RO. Variable removal of *N*-Nitrosodimethylamine (NDMA) by the RO processes could be attributed to membrane fouling and the feed water temperature. The effect of membrane fouling on *N*-Nitrosamine removal was extensively evaluated at one of the plants by conducting one month of operation and chemical cleaning of the RO element. Membrane fouling enhanced *N*-Nitrosamine removal by the pilot-scale RO process. This finding contributes to better understanding of the variable removal of NDMA by RO processes. This study also investigated the removal characteristics of *N*-Nitrosamine precursors. The NF and RO processes greatly reduced NDMA formation potential (FP), but the UF process had little effect. The contributions of MF, NF, and RO processes for reducing FPs of NDMA, *N*-Nitrosopyrrolidine and *N*-Nitrosodiethylamine were different, suggesting different size distributions of their precursors.

## 1. Introduction

In response to increasing indirect and direct potable reuse of reclaimed water, the monitoring and control of micropollutant concentrations are increasingly important. In many potable reuse schemes, reverse osmosis (RO) membranes are key components due to their high removal performance for inorganic salts and trace organic chemicals [1,2,3]. However, residual trace organic chemicals in RO permeate have been reported [4,5,6]. Among the chemicals, *N*-Nitrosamines are of key concern for potable reuse, and further research and monitoring of them are recommended [4,7,8]. These *N*-Nitrosamines include *N*-Nitrosodimethylamine (NDMA), *N*-Nitrosomethylethylamine (NMEA), *N*-Nitrosopyrrolidine (NPYR), *N*-Nitrosodiethylamine (NDEA), *N*-Nitrosopiperidine (NPIP), *N*-Nitrosomorpholine (NMOR), *N*-Nitrosodipropylamine (NDPA), and *N*-Nitrosodi-*n*-butylamine (NDBA). Most of these *N*-Nitrosamines are probable carcinogens [9], and their frequent occurrence in raw and secondary-treated wastewater [10,11,12] and reclaimed water [7,13,14,15,16,17] has been reported. For the augmentation of drinking water sources, the California Department of Public Health sets a drinking water notification level of 10 ng/L for NDMA and NDEA [18], and the Australian Guidelines for Water Recycling sets guideline values for NDMA (10 ng/L), NDEA (10 ng/L), and NMOR (1 ng/L) [19].

*N*-Nitrosamines, in particular NDMA, are of great concern in potable water reuse because they occur ubiquitously in treated wastewater and readily permeate RO membranes. Due to the low and variable removal of NDMA by RO membranes, additional processes such as UV processes and UV-based advanced oxidation processes are installed to remove NDMA from RO permeate, which results in a high energy cost. The removal characteristics of *N*-Nitrosamines by RO membranes have been extensively investigated at laboratory-scale [20,21,22,23]. Since *N*-Nitrosamines are hydrophilic and non-ionized at the typical environmental pH range of 6–8, their removal by RO membranes is governed mainly by steric hindrance. A laboratory-scale study showed increased removal of *N*-Nitrosamines in order of increasing molecular weight of *N*-Nitrosamines [21]. The previous study also found a significant decrease in the removal of *N*-Nitrosamines with increasing feed water temperature, and a discernible decrease in NDMA removal with decreasing feed water pH and with increasing feed water ionic strength. An increase in the removal of *N*-Nitrosamines with increasing permeate flux has also been observed [21]. In addition, RO membrane characteristics influence the removal of *N*-Nitrosamines [22]. Furthermore, membrane fouling can enhance NDMA removal by RO membranes as a result of enhanced size exclusion [23].

These laboratory-scale studies show the removal characteristics of *N*-Nitrosamines by RO membranes. However, pilot-scale data to assess the effects of realistic operating conditions on *N*-Nitrosamines removal are scarce. In addition, the previous pilot-scale studies have focused extensively on NDMA and evaluation for other *N*-Nitrosamines are still limited. Fujioka et al. assessed the removal of eight *N*-Nitrosamines in three full-scale RO plants and reported that discrepancies in *N*-Nitrosamine removal data between laboratory- and full-scale studies were occurred probably due to differences in water recovery and operating conditions such as temperature, membrane fouling, and hydraulic conditions [14]. The authors assessed the effects of operating conditions on *N*-Nitrosamines removal. However, no study evaluated the impact of membrane fouling on the removal of *N*-Nitrosamines by RO processes at pilot-scale. 

*N*-Nitrosamines have also attracted attention as disinfection by-products (DBPs), which are formed during the disinfection of biologically treated wastewater with chlorine or chloramines [24,25,26] as well as alternative disinfectants such as chlorine dioxide (ClO_2_) and ozone (O_3_) [27]. NDMA can be formed from compounds which can release a secondary amine, including pharmaceuticals [28], pesticides [29], cationic polymers and ion exchange resins [30], and quaternary amines used in toiletries [31]. Although some precursors convert to NDMA with molar yields of 90% [28], specific precursors responsible for significant NDMA formation have not yet been identified. *N*-Nitrosamines and their precursors are both present in domestic and industrial wastewater. Among domestic wastewater, laundry water has been reported as the most significant source of *N*-Nitrosamines and their precursors, followed by shower water and urine, while less contributions came from bathroom washbasin and kitchen sink waters [32]. Loadings of *N*-Nitrosamines and their precursors from industrial discharges are generally site-specific (e.g., anti-yellowing agents found in a wastewater in Japan [33,34]). 

In water reclamation processes using RO process, disinfectants are often added upstream of the RO process to inhibit biological fouling, and to the final water before distribution. Since polyamide RO membranes are susceptible to free-chlorine, chloramines are often used for mitigating biofouling on RO membranes. Since *N*-Nitrosamines are likely to be formed during chloramination, the addition of the disinfectants leads to the formation of *N*-Nitrosamines during membrane processes [35,36,37] and distribution [36]. To control *N*-Nitrosamine concentrations in reclaimed water, it is therefore important to understand the fate of their precursors during membrane treatment processes.

The fate of NDMA precursors in integrated membrane processes has been studied mainly at microfiltration (MF)–RO treatment plants [16,37,38] and ultrafiltration (UF)–RO treatment plants [35,38]. Even though the fate of NDMA precursors in nanofiltration (NF) process has been studied at bench scale, pilot-scale data to assess the fate of NDMA precursors by NF process are very scarce. Miyashita et al. investigated the removal of *N*-Nitrosamine precursors (i.e., dimethylamine, methylethylamine, diethylamine, and dipropylamine) in a bench-scale NF treatment and reported >98% removal of all four precursors [20]. However, evaluation of the removal efficiency of specific precursors by membrane processes is insufficient to evaluate the efficiency of removal of *N*-Nitrosamine precursors, because not all precursors have been identified. Mamo et al. assessed the fate of NDMA precursors in pilot-scale membrane bioreactor (MBR)–NF processes and found high rate of reduction of NDMA formation potential (FP) (>90%) by NF process [17]. The previous study has evaluated the fate NDMA precursors by a pilot-scale NF process, but assessment of the fate of other *N*-Nitrosamine precursors is still limited.

This study aimed: (1) to investigate the occurrence and fate of eight *N*-Nitrosamines and their FPs at three pilot-scale water reclamation plants using MF, UF, NF and RO processes, (2) to explore realistic operating conditions that contribute to the variability of the removal of *N*-Nitrosamines by pilot-scale RO processes; and (3) to extensively assess the effect of membrane fouling on the removal of *N*-Nitrosamines by a pilot-scale RO process by performing chemical cleaning of the RO element.

## 2. Materials and Methods

### 2.1. Site Description

**Figure 1:**
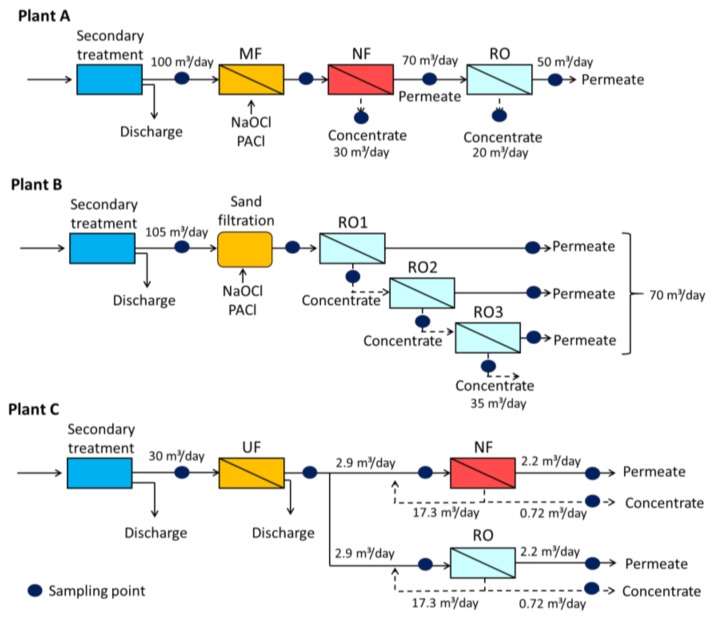
Process flows and sampling points in the water reclamation plants.

Samples were collected at three pilot-scale water reclamation plants (A, B, C) in Japan receiving secondary effluent derived from municipal wastewater treatment plants. Plant A applies an MF-NF-RO system following an anaerobic–oxic activated sludge process (Figure 1, Table 1). Plant B uses a sand filtration (SF) and three-stage RO system following a conventional activated sludge process (Figure 1, Table 1). Plant C applies UF and two parallel NF and RO systems following a conventional activated sludge process (Figure 1, Table 1). The membrane systems at the three plants were operated at a constant permeate flux condition. The operating conditions of the membrane processes are shown in Table 4 (Section 3.3). To mitigate biofouling of membranes, sodium hypochlorite was continuously added to the MF feed water at plant A and to the SF feed water at plant B. The added chlorine was converted to chloramines (Table 3) by the reaction with ammonia present in the secondary effluent (data not shown) at these plants, at which nitrification was not performed during the secondary treatment. Polyaluminum chloride (PACl) was added as a coagulant prior to the MF and SF processes. To mitigate biofouling at plant C, 2,2-dibromo-3-nitrilopropionamide was periodically added prior to the NF and RO processes.

### 2.2. Sampling Protocol

Grab samples were taken across the water reclamation treatment trains (Figure 1). All samples were collected in amber glass bottles and stored in darkness at 4 °C until analysis. Chlorine concentrations were measured with chlorine test kits (Pocket Colorimeter II, Hach, Loveland, CO, USA) at plants A and B, where sodium hypochlorite was added upstream of the MF and SF processes (Figure 1). To quench residual chlorine and stop the formation of *N*-Nitrosamines, sodium thiosulfate was added to all samples except secondary effluent to give a final concentration of 10 mg/L. Water temperature, pH, and conductivity were measured on site with a multi-function water quality meter (U-52G, Horiba, Kyoto, Japan). Operating pressures and flows were monitored within the plants. Sampling campaigns were conducted at plant A from January 2013 to August 2014 (*n* = 3), at plant B from December 2012 to December 2014 (*n* = 3), and at plant C from June 2013 to January 2014 (*n* = 5).

### 2.3. Chemical Cleaning of RO Element

To assess the effect of RO membrane fouling on removal of *N*-Nitrosamines, RO feed and permeate samples at plant C were collected at different stages of membrane fouling development. The RO process was operated at constant permeate flux condition of 21 L/m^2^ h. Samplings were conducted once a week from November to December 2015. Since feed water temperature was not so fluctuated during the sampling period (27–30 °C), TMP values monitored at each sampling campaign were used as an indicator of membrane fouling development. After 1 month of operation, the RO element was chemically cleaned as follows: (1) circulate cleaning solutions (below) in the RO system for 1 h; (2) soak the element in cleaning solution for 1 h; and (3) flush sufficient RO permeate to displace remaining cleaning solution. The chemical cleaning was performed using 0.03% sodium dodecyl sulfate (SDS) with sodium hydroxide (pH 11), 2% citric acid (pH 2), and sodium hydroxide (pH 11). The changes in TMP were monitored after each cleaning to evaluate the effectiveness of the chemical cleanings for removing membrane foulants. RO feed and permeate samples were collected before and after the cleaning to evaluate the effect of chemical cleaning on membrane fouling and removal of *N*-Nitrosamines. 

### 2.4. Analytical Techniques

#### 2.4.1. *N*-Nitrosamines

Eight *N*-Nitrosamines—NDMA, NMEA, NPYR, NDEA, NPIP, NMOR, NDPA, and NDBA—were targeted in this study. These *N*-Nitrosamines have molecular weights in the range of 74 to 158 g/mol (Table 2). An analytical method previously developed for the determination of *N*-Nitrosamines in wastewater was employed [25]. This method uses solid-phase extraction, gas chromatography and analysis by tandem mass spectrometry. Eight deuterated *N*-Nitrosamines—NDMA-*d*_6_, NMEA-*d*_3_, NPYR-*d*_8_, NDEA-*d*_10_, NPIP-*d*_10_, NMOR-*d*_8_, NDPA-*d*_14_, and NDBA-*d*_18_—were used as surrogate. These deuterated chemicals were obtained from CDN Isotopes (Pointe-Claire, QC, Canada) and a stock solution was prepared in pure methanol at 1 mg/L of each deuterated *N*-Nitrosamines. After spiking surrogate solution into each sample, *N*-Nitrosamines were extracted with Sep-Pak NH-2 and AC-2 cartridges (Waters, MA, USA) at a flow rate of 10 mL/min. After the AC-2 cartridges were dried, the analytes were eluted from the cartridges with 2 mL dichloromethane (Wako Pure Chemical Industries, Tokyo, Japan) and were concentrated under a nitrogen gas stream. After adding dichloromethane solution and deuterated toluene (toluene-*d*_8_) stock solution (Supelco, Bellefonte, PA, USA), used as an injection internal standard, into the eluents, *N*-Nitrosamine concentrations were quantified using Varian 450 Series gas chromatograph coupled with a Varian 300 series tandem mass spectrometer. The limits of detection and quantification of *N*-Nitrosamines are shown in Table S1.

#### 2.4.2. Formation Potentials

*N*-Nitrosamine precursors were evaluated as formation potentials as described [24] with minor modifications [11]. In brief, 20 mM chloramine stock solution (1400 mg Cl_2_/L) was prepared before each experiment because of its tendency to auto-decompose at high concentrations. First, the sample pH was adjusted to 7 with 10 mM phosphate buffer. Next, chloramination was performed in a 1-L amber glass bottle by adding 20 mM chloramine stock solution (100 mL) to samples (900 mL). The samples were then stirred for 10 days at room temperature on a shaker (NR-80, Taitec, Saitama, Japan). FP was defined as the increment of *N*-Nitrosamine concentration during 10 days and calculated by subtracting background *N*-Nitrosamine concentrations from the final concentration.

### 2.5. Calculations

*N*-Nitrosamine removal rate ($R_{N}$) and FP reduction rate ($R_{\mathrm{FP}}$) in each process was calculated using the following equations:

(1) $$R_{N}\;\left[\text{\%}\right]=\left(1-\frac{C_{p}}{C_{f}}\right)\times 100$$

(2) $$R_{\mathrm{FP}}\;\left[\text{\%}\right]=\left(1-\frac{FP_{p}}{FP_{f}}\right)\times 100$$

where $C_{p}$ and $C_{f}$ are *N*-Nitrosamine concentrations in the permeate and feed, respectively; and $FP_{p}$ and $FP_{f}$ are formation potentials in the permeate and feed, respectively.

## 3. Results and Discussion

### 3.1. Occurrence of N-Nitrosamines

**Figure 2:**
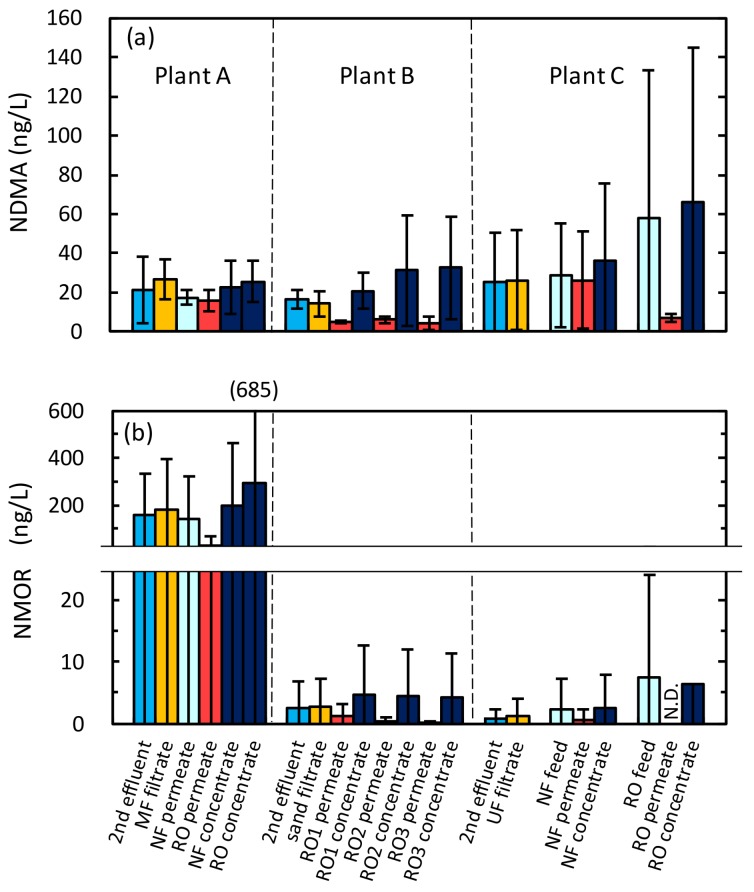
Concentrations of (**a**) NDMA and (**b**) NMOR detected across the treatment trains at plants A, B, and C (means ± standard deviation (SD)). N.D.: not detected.

NDMA and NMOR were prevalent *N*-Nitrosamines, as reported in other studies [7,13,14]. NDMA was detected in all secondary effluent samples at 9 to 41 ng/L at plant A, 11 to 20 ng/L at plant B, and 8 to 70 ng/L at plant C (Figure 2). NMOR was present in some secondary effluent samples at 50 to 358 ng/L at plant A, N.D. (not detected) to 8 ng/L at plant B, and N.D. to 4 ng/L at plant C (Figure 2). Along with NDMA and NMOR, NDBA was detected in secondary effluent at plant B in the second sampling campaign at 11 ng/L. NMEA, NPYR, NDEA, NPIP, and NDPA were not detected at any sampling campaigns.

NDMA was detected in all RO permeate samples at 12 to 22 ng/L at plant A, 2 to 8 ng/L at plant B, and 5 to 10 ng/L at plant C (Figure 2). Due to the low NDMA removal by the RO process, NDMA concentrations in RO permeate were slightly higher than the guideline value of 10 ng/L [19] at plant A, but were lower than the guideline value at plants B and C. NMOR was also detected in RO permeate samples at plants A and B. RO permeate samples at plant A had high concentrations of NMOR (7–71 ng/L), which was much higher than the guideline value of 1 ng/L established for potable reuse [19]. High NMOR concentrations in RO permeate have been reported in previous studies (5–30 ng/L [10]; 177–475 ng/L [14]). These results indicate that both NMOR and NDMA can pose risks for potable reuse and should be monitored at water reclamation plants.

### 3.2. Occurrence of Formation Potentials

**Figure 3:**
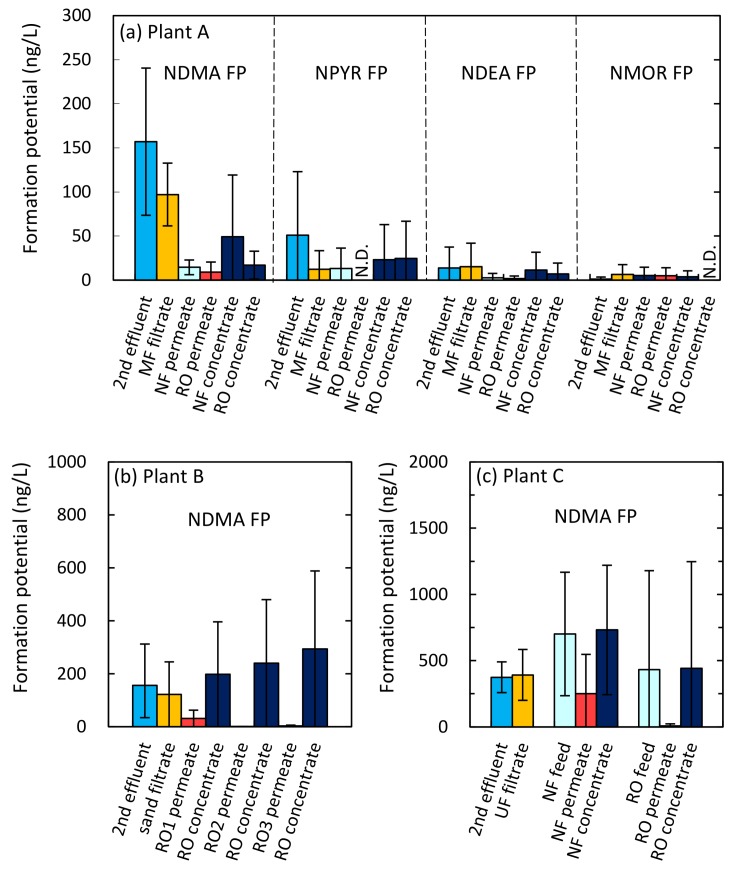
(**a**) *N*-Nitrosamine FPs found across the treatment trains at plant A, and (**b**,**c**) NDMA FPs found at plants B and C (means ± SD). N.D.: not detected.

NDMA FP was found most frequently at the three plants (Figure 3). The NDMA FP level in secondary effluent ranged from 67 to 232 ng/L at plant A, from 70 to 242 ng/L at plant B, and from 236 to 482 ng/L at plant C. These NDMA FP levels were in general lower than values reported from the U.S. (200–2000 ng/L [12]) and Australia (300–1020 ng/L [35]). In addition to NDMA FP, NPYR FP (101 ng/L), NDEA FP (41 ng/L), and NMOR FP (4 ng/L) were present in secondary effluent at plant A at the second sampling campaign (Figure 3a).

The presence of *N*-Nitrosamine FPs could lead to the formation of *N*-Nitrosamines during the membrane processes due to the reaction between precursors and disinfectants. Chlorines added upstream of the MF process at plant A and of the SF process at plant B (Figure 1) were converted to chloramines by reaction with ammonium in the secondary effluent (Table 3). In addition to the *N*-Nitrosamine FP levels and disinfectant concentrations, the contact time between disinfectant and water is an important factor affecting *N*-Nitrosamines formation. Plants A and B were pilot-scale plants and the contact times were less than 2 h.

To evaluate the formation of *N*-Nitrosamines during the membrane processes, mass loadings of *N*-Nitrosamines across the treatment train at plant A were calculated. There was no increase in NDMA mass loadings during the NF and RO processes (Figure S1). This result indicates that NDMA was not formed during these processes despite the presence of NDMA precursors and chloramines, which could be resulted from the relatively low chloramine concentrations (Table 3) and short contact time between water and chloramines during the NF and RO processes (<30 min). On the other hand, NDMA mass loadings increased during the MF process in the second sampling campaign, indicating NDMA formation (Figure S1). At the second sampling campaign at plant A, relatively high chloramine concentration (1.2 mg/L) was observed, which could explain the NDMA formation. Since NDMA was not well removed by the RO process, some NDMA formed during the MF process may pass through the RO membrane and increase NDMA concentration in the final product water. The chlorine concentration added needs to be optimized to avoid the formation of excessive NDMA in the treatment process. NDEA, NPYR, and NMOR were not formed across the treatment train despite the presence of FPs.

NDMA FP was also found in some RO permeate samples at 2 to 22 ng/L at plant A, N.D. to 89 ng/L at plant B, and N.D. to 29 ng/L at plant C (Figure 3). These values are comparable to values reported from the U.S. (12–59 ng/L [12]) and Australia (6 ng/L [35]). Even though NDMA FPs in RO permeate were lower than those in secondary effluent, NDMA precursors remaining in RO permeate might reduce RO permeate quality. For example, a previous study reported an increase in NDMA concentration from 13 ng/L to 24 ng/L during 24 h chloramination of RO permeate [36]. In assessing NDMA risks associated with the use of reclaimed water, not only NDMA but also NDMA FP in RO permeate need to be considered.

### 3.3. Removal of N-Nitrosamines

**Figure 4:**
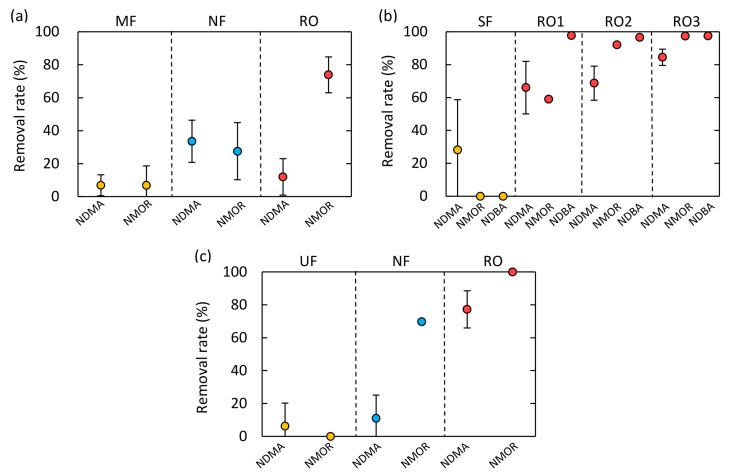
*N*-Nitrosamines removal by each membrane process at (**a**) plant A, (**b**) plant B, and (**c**) plant C (means ± SD). Error bars are not shown for the compounds detected once.

**Figure 5:**
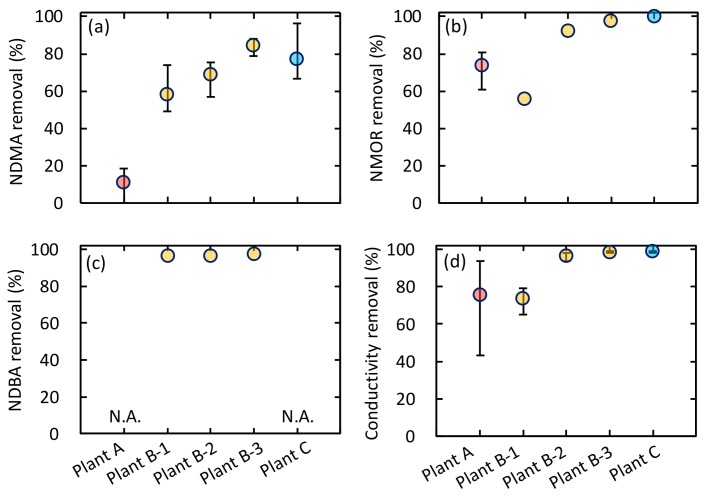
Removal of (**a**) NDMA, (**b**) NMOR, (**c**) NDBA, and (**d**) conductivity by RO processes at plants A, B, and C (means ± minimum and maximum). N.A.: not available. Error bars are not shown for the compounds detected once.

The three integrated membrane systems provided variable removal performance for the detected three *N*-Nitrosamines (Figure 4). Although the RO processes removed NMOR and NDBA with high efficiency, their removal of NDMA varied significantly, even at the same plant (Figure 5). The different removal rates of the three *N*-Nitrosamines can be attributed to their molecular weight because *N*-Nitrosamines removal by RO membranes is governed mainly by steric hindrance.

#### 3.3.1. Plant A

The MF process exhibited limited removal for NDMA (<8%) and NMOR (<20%) (Figure 4a). The small NDMA molecule easily passes through the MF process, and the removal level was comparable to a previous report of <10% [39]. The NF process gave low to moderate removal rate for efficiency for NDMA (13–41%) and NMOR (16–47%), respectively (Figure 4a). The rates of NDMA removal in our study (13–41%) were slightly higher than those reported in previous studies (<10% [22]; <20% [20]). Since enhanced NDMA removal by fouled NF membranes was reported in a previous study [40], the relatively high NDMA removal observed in this study might relate to membrane fouling.

The difference in removal efficiency between NDMA and NMOR was noticeable during the RO process (Figure 4a). While the removal of NDMA was <19%, that of NMOR was as high as 81%. This result is in accordance with a previous study reporting that the removal of *N*-Nitrosamines by RO membranes increased in order of increasing molecular weight [21]. Thus, molecular size can significantly affect removal by RO processes. Surprisingly, the RO process exhibited lower removal rate for NDMA (<19%) than the NF process (19–41%) at plant A. Since the RO process exhibited low removal rates for conductivity (Figure 5d), the quite low NDMA removal can be attributed to damage of the RO membrane rather than to membrane characteristics or operational conditions. The RO membrane applied at plant A is a polyamide membrane, which is susceptible to free chlorine attack. Chlorine can destruct polymer structure of RO membranes and cause an increase in free volume and flexibility of the polymer chain [41]. Even though sodium hypochlorite added to the MF feed water at plant A (Figure 1) was mostly converted to chloramines (Table 3), the residual free chlorine might have caused the RO membrane damage. Since *N*-Nitrosamines removal by RO membranes are governed mainly by steric hindrance effect, the enlargement of free-volume hole-size of damaged membrane can decrease the removal of *N*-Nitrosamines, in particular the removal of low molecular weight *N*-Nitrosamines (i.e., NDMA). 

#### 3.3.2. Plant B

As expected, removal of NDMA, NMOR, and NDBA by sand filtration was limited, indicating that these *N*-Nitrosamines were too small to remove by sand filtration (Figure 4b). Plant B applies a three-stage RO process following the sand filtration. The removal of *N*-Nitrosamines by the three RO stages ranged from 49 to 88% of NDMA, from 59 to 97% of NMOR, and >97% of NDBA. The rates of removal increased in order of increasing molecular weight, as found at plant A and in other studies [20,21]. An increase in NDMA removal from the first to the third RO stage was observed (Figure 5a), which might be attributed to membrane fouling. According to laboratory-scale studies, increased NDMA removal is caused by decreasing feed water temperature, increasing permeate flux, and formation of membrane fouling [21,23]. Since the feed water temperature was the same in each RO stage (Table 4), feed water temperature was not the reason for the increased NDMA removal. Although the permeate flux of each RO stage was not monitored, permeate flux would decrease with advancing stage because each stage received the concentrate of the previous stage, and the increased salt concentration (Table 4) would result in increased osmotic pressure. A decrease in permeate flux would result in a decrease in NDMA removal [21], but this is the opposite trend observed in our study. Thus, changes in permeate flux through the three-stage RO process is not the main reason for the increased NDMA removal. On the other hand, RO membrane fouling would be more severe with advancing RO stage because each RO stage received concentrate of the previous RO stage. Enhanced removal of neutral and hydrophilic solutes by fouled RO membranes have been found in several studies [23,42,43]. The previous work suggested that membrane foulants caused a shrinking of membrane pores and prevented solute permeation through the fouled RO membranes, which resulted in increased solute removal. Based on the results from the previous studies, RO membrane fouling could attribute to the increased NDMA removal observed in this study. Water reclamation plants often apply multiple-stage RO systems to increase water recovery rate. Due to the effect of membrane fouling, variable NDMA removal can be provided in multiple-stage RO systems.

#### 3.3.3. Plant C

**Figure 6:**
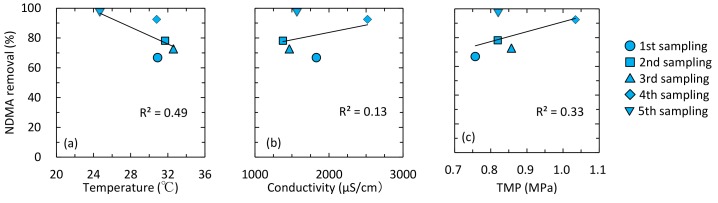
Correlations between NDMA removal and (**a**) feed water temperature, (**b**) conductivity, and (**c**) TMP. TMP values compensated with feed water temperature are shown.

Removal of NDMA and NMOR by the UF process was limited (Figure 4c). The NF process removed <32% of NDMA and 70% of NMOR. The RO process removed 67 to 96% of NDMA and >99% of NMOR. Since the RO process was operated with stable water recovery rate (75%) and permeate flux (11 L/m^2^/h), the variable NDMA removal by the RO process would be due to differences in RO feed water characteristics or to RO membrane fouling (Table 4). NDMA removal had moderate correlations with feed water temperature (*R*^2^ = 0.49; Figure 6a) and transmembrane pressure (TMP) (*R*^2^ = 0.33; Figure 6c). The TMP values were compensated with feed water temperature. An increase in NDMA removal was observed with increasing TMP (Figure 6c), with the only exception in the fifth sampling campaign. This result suggests that membrane fouling is a key factor enhancing NDMA removal by the RO process. The effect of membrane fouling on *N*-Nitrosamines removal by RO process was extensively evaluated by performing 1 month of operation and chemical cleaning of the RO element (discussed in Section 3.4). In the fifth sampling campaign, high NDMA removal was observed regardless of the relatively low TMP value (Figure 6c). The high NDMA removal might be attributed to feed water temperature. According to previous study, decreased feed water temperature resulted in an increase in NDMA removal by RO membranes [21]. One possible reason for this trend is a decrease in NDMA permeability through the RO membrane, which are resulted from increased water viscosity and decreased solute diffusivity. Another possible reason is a shrink of free-volume hole-size of the RO membrane. Decreased feed water temperature can affect the polymer structure of RO membranes and decrease free-volume hole-size [44]. For these reasons, feed water temperature could be the reason for the high NDMA removal.

### 3.4. Impact of RO Membrane Fouling on N-Nitrosamines Removal

**Figure 7:**
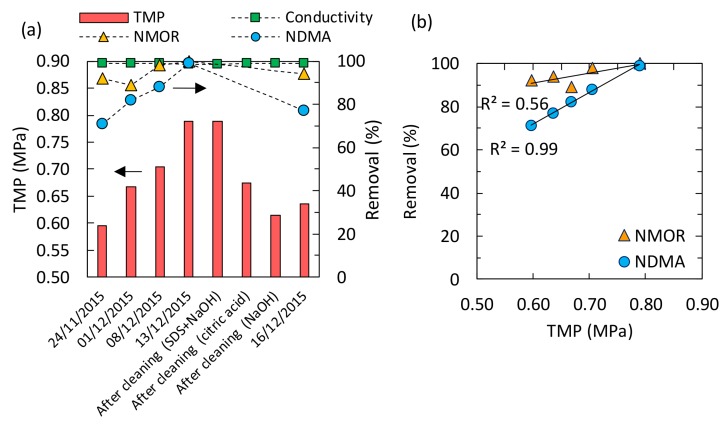
(**a**) Relations between TMP and removal of NDMA, NMOR, and conductivity before and after chemical cleaning (*n* = 1), and (**b**) correlation between TMP and removal of the two *N*-Nitrosamines.

To assess the impact of RO membrane fouling on *N*-Nitrosamines removal, samplings were conducted at plant C every week for 1 month. During 1 month of operation, TMP increased from 0.60 to 0.79 MPa, indicating the development of RO membrane fouling (Figure 7a). At the same time, NDMA removal increased from 71 to 99% and NMOR removal increased from 92 to 99% (Figure 7a). After the 1 month of operation, chemical cleaning was conducted using (1) 0.03% SDS and sodium hydroxide (pH 11), (2) 2% citric acid (pH 2), and (3) sodium hydroxide (pH 11). When the RO element was cleaned with SDS and sodium hydroxide, TMP value was kept at 0.79 MPa (Figure 7a), indicating the cleaning solution had no impacts on removing membrane foulants. On the other hand, TMP value was decreased from 0.79 to 0.68 MPa by the cleaning with citric acid, and further decreased to 0.62 MPa with sodium hydroxide (Figure 7a), indicating partial reduction of membrane fouling. Since citric acid and sodium hydroxide solutions are effective to remove inorganic and organic foulants, respectively, the reduction of membrane fouling by these chemical solutions suggests the presence of inorganic and organic foulants on the RO element.

After performing these chemical cleanings, NDMA removal decreased from 99 to 77% and NMOR removal decreased from 99 to 94%. NDMA removal had a strong correlation with TMP (*R*^2^ = 0.99, *n* = 5; Figure 7b). Feed water temperature and permeate flux, which have been reported to affect NDMA removal by RO membranes, did not fluctuate enough to affect NDMA removal during the sampling campaigns (Table 5). These results indicate that membrane fouling enhanced the removal of these *N*-Nitrosamines. In a laboratory-scale study, membrane fouling increased the removal of micropollutants by RO membrane by enhancing the effect of size exclusion [23]. Our data indicate that RO membrane fouling is one of the most important factors affecting *N*-Nitrosamines removal, even at pilot scale.

### 3.5. Rate of Reduction of N-Nitrosamine FPs

**Figure 8:**
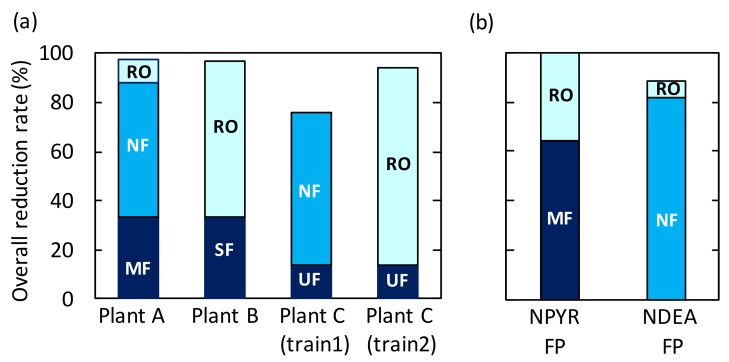
Overall rates of reduction of (**a**) NDMA FP at plants A, B, and C and of (**b**) FPs of NPYR and NDEA at plant A.

The overall reduction rates of NDMA FP achieved was more than 75% at the three plants (Figure 8a). Even though NDMA was not sufficiently removed by the NF and RO processes, precursors of NDMA were well removed by these processes. This result suggests that most precursors were ionized or were large enough to be removed by the NF and RO processes. However, the reduction rates of NDMA FP by the NF processes were lower than values (>98%) reported in a previous study which investigated the removal of four NDMA precursors (dimethylamine, ethylmethylamine, diethylamine, and dipropylamine) using a bench-scale NF process [20]. That study attributed the high rates of removal to electrostatic interactions between the negatively charged NF membrane and the positively charged precursors. Therefore, the lower rate of reduction by the NF processes observed in our study suggests that some NDMA precursors were present in their uncharged forms and were thus difficult to remove by the NF processes. For example, dimethylsulfamide and dimethylformamide are small uncharged NDMA precursors which can likely pass through NF processes [38]. Further research to identify uncharged NDMA precursors and their efficiencies of removal by water reclamation processes is necessary.

While NF and RO processes at the three plants removed high proportions of NDMA FP, the MF and SF processes removed widely variable proportions: <59% by MF at plant A and <67% by SF at plant B (Figure 8a). The relatively high reduction rates by the MF and SF processes could be attributed to the effect of coagulant added upstream. Wang et al. reported that coagulation preferentially removed NDMA precursors with molecular weight >30 kDa and hydrophobic fraction of a biologically treated wastewater [45]. Charge neutralization of negatively charged colloids by coagulant might be attributed to adsorption of the NDMA precursors onto some colloids or particulate compounds, which could be the reason for the reduction of NDMA FP due to the addition of coagulant. On the other hand, the UF process at plant C removed little NDMA FP (Figure 8a). These results suggest that NDMA precursors were present mainly as dissolved compounds at plant C, but partially as colloidal or particulate compounds at plants A and B.

FPs of NPYR, NDEA, and NMOR were detected only at plant A. Since NMOR FP was low (Figure 3a), and it was difficult to clearly evaluate reduction across the treatment train, the efficiency of the integrated membrane processes was evaluated only for FPs of NPYR and NDEA. These FPs were effectively reduced (>89%) by the MF-NF-RO system (Figure 8b). The membrane processes were effective at removing these *N*-Nitrosamine precursors. However, the efficiencies of removal of the *N*-Nitrosamine FPs differed among processes, indicating different size distributions of these *N*-Nitrosamine precursors. For example, the contribution of each membrane process to the overall rates of reduction of NPYR FP were 64% by MF, 1% by NF, and 36% by RO, and those of NDEA FP were negligible by MF, 82% by NF, and 7% by RO (Figure 8b). Most NPYR precursors were removed by the MF process, and the rest were removed mostly by the RO process. This result suggests that most NPYR precursors were present as colloidal or particulate compounds and rest were present as dissolved compounds, which cannot be removed well by the NF process. On the other hand, NDEA precursors were removed not by the MF process, but by the NF and RO processes. This result suggests that NDEA precursors would be predominantly present as dissolved compounds.

## 4. Conclusions

This study investigated the removal characteristics of *N*-Nitrosamines and their precursors at three pilot-scale membrane systems for water reclamation. The main reasons for the variable NDMA removal by the RO process at a plant could be membrane fouling and feed water temperature. The effect of membrane fouling on *N*-Nitrosamines removal by an RO process was extensively evaluated at one of the plant by conducting 1 month of operation and chemical cleaning of the RO element. The extensive evaluation at a plant revealed that membrane fouling enhanced *N*-Nitrosamines removal by the pilot-scale RO process. The finding contributes to better understanding of the variable NDMA removal by RO processes. The three-stage RO process at a plant showed increased NDMA removals as progress of the RO stages, which might be attributed to the effect of membrane fouling. Since RO membranes in multi-stage RO system would have different membrane fouling or permeate flux conditions, these differences might lead to variable NDMA removals by multi-stage RO processes. 

This study also investigated the removal characteristics of *N*-Nitrosamine precursors at the three plants. NDMA FP was predominant at all three plants. The NF and RO processes were effective to reduce NDMA FP. However, remaining NDMA FP in their permeates implies the presence of uncharged precursors that are not easily removed by the membrane processes. FPs of NPYR, NDEA, and NMOR were present at a plant which used MF-NF-RO system. Each membrane process reduced FPs of NPYR and NDEA with different efficiencies, suggesting different size distributions of their precursors. In designing new water reclamation plants, it is important to investigate which kinds of *N*-Nitrosamine FPs are present in the feed water.

## Figures and Tables

**Table 1:** Characteristics of membranes used at plants A, B, and C.

Plant	Membrane	Membrane Material	Type	Pore Size ^a^ (µm)	NaCl Rejection ^a^	Element Size	Number of Elements Per Vessel	Number of Vessels Per Unit	Recovery Rate (%)
A	MF	Ceramic	Cylindrical	0.1	N.A.	N.A.	N.A.	N.A.	100
	NF	Poly-vinyl alcohol polyamide	Spiral	N.A.	92%	4 inch	1	1	65
	RO	Fully aromatic polyamide	Spiral	N.A.	99.5%	4 inch	1	1	80
B	RO	Aromatic composite polyamide	Spiral	N.A.	99.7%	4 inch	1	7	67
C	UF	Poly-vinylidene fluoride	Hollow fiber	0.01	N.A.	N.A.	N.A.	N.A.	100
	NF	Piperazine polyamide	Spiral	N.A.	60%	4 inch	1	1	75
	RO	Aromatic composite polyamide	Spiral	N.A.	99.7%	4 inch	1	1	75

N.A.: not available. ^a^ Pore size and NaCl rejection values as specified by the manufacturers.

**Table 2:** Physicochemical properties of eight *N*-Nitrosamines.

Compound	NDMA	NMEA	NPYR	NDEA	NPIP	NMOR	NDPA	NDBA
Molecular weight (g/mol) ^a^	74.08	88.11	100.12	102.14	114.15	116.12	130.19	158.25
$pK_{a}$ ^a^	3.22	3.42	3.30	3.32	3.30	3.14	3.30	3.30
$LogK_{ow}$ ^a^	0.08	0.41	0.39	0.75	0.81	−0.32	1.05	2.56

^a^ Calculated in MarvinSketch software (ChemAxon, Budapest, Hungary).

**Table 3:** Concentration of chloramines across the treatment trains of plant A and plant B.

Sampling Campaign	Plant A				Plant B						
	Concentration (mg/L)				Concentration (mg/L)						
	NF Perm.	NF Conc.	RO Perm.	RO Conc.	Sand Filter Eff.	1st RO Perm.	1st RO Conc.	2nd RO Perm.	2nd RO Conc.	3rd RO Perm.	3rd RO Conc.
1st	0.33	0.29	0.20	0.31	1.03	0.41	0.65	0.30	0.81	0.38	0.81
2nd	1.18	1.15	1.18	1.20	0.98	0.19	0.35	<0.02	0.40	<0.02	0.46
3rd	0.03	0.09	<0.02	0.02	3.24	0.42	0.67	0.21	0.39	0.13	0.33

**Table 4:** Operating conditions and feed water qualities of the NF and RO processes.

Plant	Sampling Campaign	Membrane Process	Permeate Flux (L/m² h)	TMP (MPa) ^a^	Feed Temperature (°C)	Feed pH	Feed Conductivity (µS/cm)
A	1st	NF	24.8	0.41	16.1	8.1	815
		RO	24.8	0.48	16.2	7.9	579
	2nd	NF	24.8	0.38	16.5	7.8	765
		RO	24.8	0.48	16.7	7.1	515
	3rd	NF	24.8	0.34	29.2	7.0	867
		RO	24.8	0.44	29.5	7.1	577
B	1st	1st-stage RO	N.A.	N.A.	18.3	5.3	582
		2nd-stage RO	N.A.	N.A.	18.9	5.9	739
		3rd-stage RO	N.A.	N.A.	19.3	5.2	880
	2nd	1st-stage RO	N.A.	N.A.	25.3	6.5	1230
		2nd-stage RO	N.A.	N.A.	25.7	5.1	1290
		3rd-stage RO	N.A.	N.A.	26.0	6.5	1830
	3rd	1st-stage RO	N.A.	N.A.	25.3	5.9	739
		2nd-stage RO	N.A.	N.A.	25.7	6.5	1230
		3rd-stage RO	N.A.	N.A.	26.0	6.5	1830
C	1st	RO	11.3	0.54	30.9	7.4	1830
	2nd	RO	11.3	0.57	31.7	N.A.	1377
	3rd	RO	11.3	0.58	32.6	N.A.	1465
	4th	RO	12.8	0.74	30.8	7.4	2520
	5th	RO	11.3	0.71	24.7	7.4	1570

N.A.: not available. ^a^ TMP values not compensated with feed water temperature.

**Table 5:** Operating conditions and feed water qualities of the RO process.

Sampling Campaign	Membrane Process	Water Recovery (%)	Permeate Flux (L/m²h)	TMP (MPa) ^a^	Feed Temperature (°C)	Feed Conductivity (µS/cm)	Feed TOC (mg/L)
1st	RO	57	21.5	0.60	29.9	1600	13.9
2nd	RO	56	21.0	0.67	28.4	1560	16.0
3rd	RO	55	20.7	0.70	27.4	1670	18.0
4th	RO	57	21.2	0.79	27.3	1650	14.6
5th	RO	56	21.1	0.64	26.6	1730	14.1

^a^ TMP values not compensated with feed water temperature.

## Supplementary Materials

The following are available online at http://www.mdpi.com/1660-4601/15/9/1960/s1, Figure S1: Formation rates of (a) NDMA and (b) NMOR across the membrane treatment at plant A, Table S1: Limit of detection (LOD) and limit of quantification (LOQ) for eight *N*-Nitrosamines.
