# Supplementary material for: Removal Characteristics of N-Nitrosamines and Their Precursors by Pilot-Scale Integrated Membrane Systems for Water Reuse
Source: Int J Environ Res Public Health. 2018 Sep 7;15(9):1960. doi: 10.3390/ijerph15091960 (PMC6163456; doi:10.3390/ijerph15091960)
Supplement: Supplementary file 1 [file ijerph-15-01960-s001.pdf]

Removal Characteristics of *N*-nitrosamines and Their Precursors by Pilot-scale Integrated Membrane Systems for Water Reuse

Haruka Takeuchi *, Naoyuki Yamashita, Norihide Nakada and Hiroaki Tanaka

Table S1. Limit of detection (LOD) and limit of quantification (LOQ) for eight *N*-Nitrosamines.

	NDMA	NMEA	NPYR	NDEA	NPIP	NMOR	NDPA	NDBA
LOD (ng/L)	0.1	0.4	0.4	1.0	0.5	1.0	0.9	0.9
LOQ (ng/L)	0.4	1.3	1.3	3.2	1.5	3.3	2.9	2.9

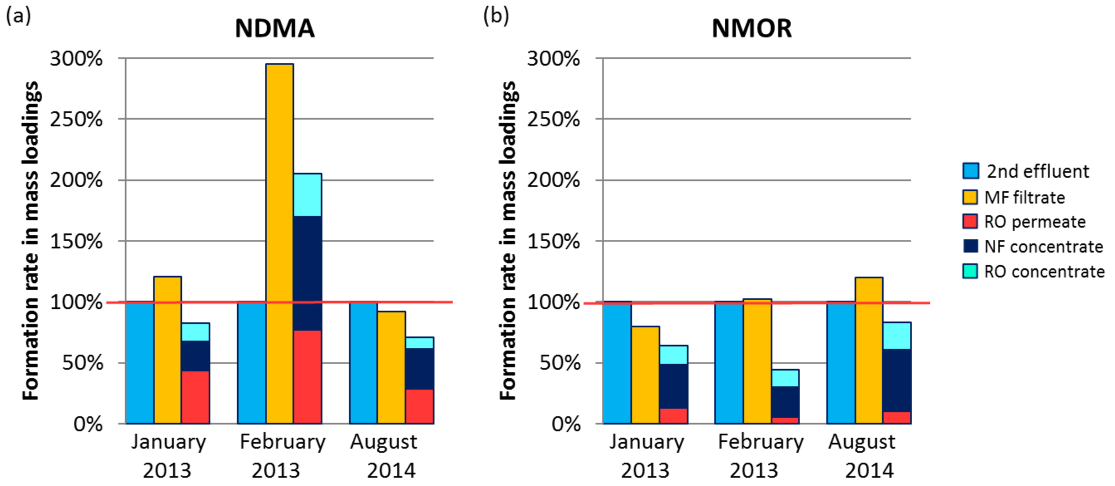

Figure S1. Formation rates of (a) NDMA and (b) NMOR across the membrane treatment at plant A.
